# It's not always Occam's razor: The pivotal role of telemedicine in stroke patients amidst the COVID-19 pandemic

**DOI:** 10.1016/j.amsu.2022.103528

**Published:** 2022-03-28

**Authors:** Talal Almas, Maryam Ehtesham, Uzair Malik, Vikneswaran Raj Nagarajan, Mahnoor Sukaina, Norah Alshareef, Ahlam Alzahrani, Suliman Abuhaimed, Yohan Porus Irani, Enaam Alzadjali, Eissa Sultan Alwheibi, Mhmod Kadom, Saif Khalid, Muhammad Shehryar, Khalid Muhammad Al Shumrani

**Affiliations:** a Royal College of Surgeons in Ireland, Dublin, Ireland; b Karachi Medical and Dental College, Karachi, Pakistan; c Imam Abdulrahman Bin Faisal University— College of Medicine, Saudi Arabia; d King Edward Medical University, Lahore, Pakistan; e Al Baha Medical College, Al Baha, Saudi Arabia

The cost of healthcare in the US grew exponentially between the years 1970 and 2016, with a documented increase of 0.243% per year on average [1]. In 2007, at the inception of the era of crippling economic, the economy witnessed a 17.7% cost increase by 2019 [2]. Contributing to this upward trend of healthcare expenditure was the advent of telemedicine. On one hand, these disruptive technologies have positively correlated with healthcare expenditure. Contrary to this, however, it is important to factor the proportion of people that may not be able to afford healthcare overall as a consequence of increased inflation [1]. These figures arose significantly since the start of the COVID-19 pandemic, which has overwhelmed and crippled healthcare systems globally. Nevertheless, it is in the midst of the pandemic that remote measures such as telemedicine are most needed.

The COVID-19 pandemic has adversely affected the entire world. Not only has it caused an unprecedented burden on healthcare systems, but it also holds responsible for inducing major economic shifts [3,4]. The challenges of living in a pandemic have given birth to various mechanisms of adaptation, such as the advent of remote working across most fields [5]. Telemedicine is the medical alternative to this. Although it has been a known entity throughout healthcare from times prior to the pandemic, the pertinence of telemedicine has been highlighted in recent times more than ever. With hospitals incurring exorbitant shortages of resources, acute patients suffering from fatal illnesses continue to lose access to priority healthcare. Moreover, the pandemic has brought to effect another medical and ethical dilemma of delineating a hierarchy or algorithm of patients requiring prompter treatment and evaluation. This has contributed to an additional burden of indecisiveness as well as the possibility to unintentionally do harm to patients in scenarios where healthcare workers are compelled to make decisions that are not fully informed. This raises yet another ethical dilemma of perhaps violating one of the cornerstone principles of medicine— non-maleficence, or “do no harm”.

Stroke is a medical presentation in which timely responses are quintessential to preventing death and disability [6]. The appropriate management of acute stroke involves immediate clinical assessment with adjunctive imaging to establish a timely diagnosis, which in turn governs decision-making for the recommended therapeutic interventions. Stroke emergencies often involve intervention with a tissue plasminogen activator (tPA) [7,8]. A recent study identified that various stroke units now employ thrombolysis routinely as well. Early administration of tPA within a 6-h window for ischemic pathology involving the anterior circulation of the brain and within a 12-h window for lesions of the posterior circulation is proven to be a rapid and effective framework that significantly reduces morbidity and mortality in stroke patients [8].

Regular hospital visits can exponentially upswing the risk of COVID-19 infection during times of the pandemic [9]. The objective here is to treat stroke patients while attempting to curb the risk of such by providing healthcare workers with the opportunity to benefit from virtual tools for assessment and treatment. Numerous technology companies provide state-of-the-art machines that have made the virtual consultation experience convenient for both clinicians and patients alike. These possibilities lend to a debate on the effectiveness in providing high quality inpatient and outpatient telemedicine services within the aforementioned cohort.

Telemedicine bridges the gap between clinicians and patients in a manner that enables provision of healthcare via application of telephonic or video consultation technologies. Telestroke is a branch of teleneurology that assists neurologists and stroke specialists to promptly intervene in events of stroke emergencies [10]. The apprehension of possible COVID-19 infection plays a pivotal role in patient presentation to hospitals during such times. The psychological impact elicited by physically attending hospital emergency departments renders telemedicine a trusted, safe and secure platform for patients to address their healthcare concerns. Although telemedicine has its obvious limitations, it preserves patient continuity of care and prevents a total lack of communication between patient and the physicians in the midst of the pandemic. Furthermore, it successfully reduces the healthcare burden of COVID-19, especially from a hospital-associated infection perspective. This includes a further advantage of preservation of the limited amount of personal protective equipment (PPE) available [11].

The evaluation of acute stroke involves immediate acquisition of the relevant radiological diagnostic modalities, such as computed tomography (CT) brain scans or access to magnetic resonance imaging (MRI) facilities. These are extremely valuable entities in stratifying the type of stroke along with the interventions that it necessitates [12]. The American Heart Association/American Stroke Association (recommendation 1, level B.) has emphasized the significance of digital review of scans by means of the Food and Drug Administration [12]. Digital reviewing of CT and MRI scans by the Alberta Stroke Program Early CT Score (ASPECTS) and Artificial Intelligence Algorithm have divulged better results than conventional methodologies [10]. This detects acute lesions as well as initial ischemic changes. Therefore, rapid diagnosis is more likely by this digital approach, which correlates with better clinical outcomes as a consequence of prompter therapeutic intervention [10].

Televideo consultation provides stroke care via initial triage and assessment of severity through the NIH stroke scale (NIHSS). Moreover, televideo consultation has more significance than telephonic consultation for obvious reasons [[13], [14], [15]]. For stable patients on chronic treatment, telemedicine provides medication refills for patients without long wait times in hospital or clinic lobbies [16]. A study by Walsh et al. compares the outcomes of virtual communication and in-person consultation for CADASIL patients [17]. Fifty patients attended the clinic, while sixty-four patients used telemedicine services. A post-visit survey concluded satisfactory results for the telemedicine service. This further champions the advantages of telemedicine as equivalent to face-to-face consultation especially in times of COVID-19 [18].

Studies report the incidence of acute stroke as a neurological manifestation in patients with COVID-19 infection. Younger patients experience a severe degree of stroke corresponding to large vessel occlusion with multiple foci involvement. Older patients are prone to developing stroke in the light of coexisting cardiovascular disease. A study reports a 7.6-fold increase in the incidence of stroke in COVID-19 patients when compared to stroke prevalence in influenza infection [19]. However, the impact that multiple organ failure plays in COVID-19 patients could possibly underpin the pathophysiology of the increased prevalence of acute ischemic stroke events that contributes to increased mortality in these patients. Newly introduced protocols require patients to undergo mandatory COVID-19 screening prior to interventional radiology angiography, which tends to delay the golden hour of receiving the triage protocol for stroke [20]. A study of 829 cases by Nannoni et al. exhibits that 44.7% of strokes in COVID-19 patients tend to be cryptogenic and acute ischemic in nature with only 3.3% of strokes being due to small artery involvement. Moreover, cardioembolic stroke was reported in 21.9% of patients, followed by 10.6% atherosclerotic large vessel stroke. Existing comorbidities increase the severity of acute stroke symptoms that lead to large vessel thromboembolism [19]. When new waves of COVID-19 outbreaks compel physicians across all specialties to tend to both their regular responsibilities as well as play a role as frontline workers for COVID-19, the risk of in-hospital permeation of COVID-19 and hospital-acquired COVID-19 increases immensely. Therefore, to further support the argument, a study by Sasaki et al. and Gordian J. et al. conclude that telemedicine is an ideal alternative to in-hospital consultation to access healthcare whilst decreasing the spread of COVID-19 infection. Telemedicine also reduces the door-to-needle time in acute stroke events and thus demonstrates a superior system of quality control [21,22].

New opportunities give rise to novel challenges. For example, a significant proportion of elderly patients find it an arduous task to operate the technological gadgets for accessing telemedicine consultations. Similarly, populations of lower socioeconomic status may not be able to afford the electronic devices required to access telehealth [17]. That said, it is most practical to cater to stroke emergencies by aiming to curb the risk of contracting COVID-19 infection. Amid the advent of disruptive innovations in the current pandemic and post-pandemic era, the telemedicine facility provides ease for continuous inpatient care. It is imperative that the telemedicine approach to the management of acute stroke emergencies becomes a formally taught niche of clinical training for stroke specialists in order to enable the safe application of this technology in the emergency setting.

## Ethical approval

NA.

## Sources of funding

This research did not receive any specific grant from funding agencies in the public, commercial, or not-for-profit sectors.

## Author contribution

TA, ME, UM: conceived the idea, designed the study, and drafted the manuscript., VRN, MS, NA, AA: conducted literature search and created the illustrations., SA, YPI, SK,: revised the manuscript critically and refined the illustrations., ESA, MA, MS, EA, KMAS: revised the final version of the manuscript critically and gave the final approval.

## Registration of research studies

1. Name of the registry: NA
2. Unique Identifying number or registration ID: NA
3. Hyperlink to your specific registration (must be publicly accessible and will be checked): NA

## Guarantor

Talal Almas, RCSI University of Medicine and Health Sciences, 123 St. Stephen's Green, Dublin 2, Ireland.

## Consent

NA.

## Provenance and peer-review

Not commissioned, externally peer-reviewed.

## Declaration of competing interest

NA.
